# A Novel Homozygous Variant in the Fork-Head-Associated Domain of Polynucleotide Kinase Phosphatase in a Patient Affected by Late-Onset Ataxia With Oculomotor Apraxia Type 4

**DOI:** 10.3389/fneur.2019.01331

**Published:** 2020-01-15

**Authors:** Rosa Campopiano, Rosangela Ferese, Fabio Buttari, Cinzia Femiano, Diego Centonze, Francesco Fornai, Francesca Biagioni, Maria Antonietta Chiaravalloti, Mauro Magnani, Emiliano Giardina, Anna Ruzzo, Stefano Gambardella

**Affiliations:** ^1^IRCCS Neuromed, Pozzilli, Italy; ^2^Dipartimento di Medicina dei Sistemi, Università di Roma Tor Vergata, Rome, Italy; ^3^Department of Translational Research and New Technologies in Medicine and Surgery, University of Pisa, Pisa, Italy; ^4^Department of Biomolecular Sciences, University of Urbino “Carlo Bo”, Urbino, Italy; ^5^Department of Biomedicine and Prevention, University of Rome “Tor Vergata”, Rome, Italy; ^6^Molecular Genetics Laboratory UILDM, Santa Lucia Foundation, Rome, Italy

**Keywords:** ataxia with oculomotor apraxia, *PNKP* gene, clinical exome, late onset, neurogenetics

## Abstract

Ataxia with oculomotor apraxia (AOA) is a clinical syndrome featuring a group of genetic diseases including at least four separate autosomal-recessive cerebellar ataxias. All these disorders are due to altered genes involved in DNA repair. AOA type 4 (AOA4) is caused by mutations in DNA repair factor polynucleotide kinase phosphatase (*PNKP)*, which encodes for a DNA processing enzyme also involved in other syndromes featured by microcephaly or neurodegeneration. To date, only a few AOA4 patients have been reported worldwide. All these patients are homozygous or compound heterozygous carriers for mutations in the kinase domain of *PNKP*. In this report, we describe a 56 years old patient affected by AOA4 characterized by ataxia, polyneuropathy, oculomotor apraxia, and cognitive impairment with the absence of dystonia. The disease is characterized by a very late onset (50 years) when compared with other AOA4 patients described so far (median age of onset at 4 years). In this proband, Clinical Exome Analysis through Next Generation Sequencing (NGS) consisting of 4,800 genes, identified the *PNKP* homozygous mutation p.Gln50Glu. This variant, classified as a likely pathogenic variant according to American College of Medical Genetics (ACMG) guidelines, does not involve the kinase domain but falls in the fork-head-associated (FHA) domain. So far, mutations in such a domain were reported to associate only with a pure seizure syndrome without the classic AOA4 features. Therefore, this is the first report of patients carrying a mutation of the FHA domain within the *PNKP* gene which expresses the clinical phenotype known as the AOA4 syndrome and the lack of any seizure activity. Further studies are required to investigate specifically the significance of various mutations within the FHA domain, and it would be worth to correlate these variants with the age of onset of the AOA4 syndrome.

## Introduction

Mutations in the DNA repair factor polynucleotide kinase phosphatase (*PNK*P) have been linked to multiple distinct human inherited syndromes. These include (i) microcephaly with seizures (MCSZ, MIM 613402, also known as early infantile epileptic encephalopathy-10), which is characterized by microcephaly, early-onset, intractable seizures, and developmental delay (1), (ii) progressive cerebellar atrophy and polyneuropathy (2), and (iii) ataxia with oculomotor apraxia type 4 (AOA4) (3, 4).

Ataxia with oculomotor apraxia (AOA), is a group of at least four autosomal-recessive cerebellar ataxia syndromes. They are related to genes which provide instructions for making proteins that are involved in repairing damaged DNA: (i) *APTX* (aprataxin), responsible for AOA1 (MIM 208920), a progressive syndrome associated with hypoalbuminemia and elevated levels of cholesterol (5–7); (ii) *SETX* (senataxin), responsible for AOA2 or *SCAR1* (MIM 606002), a progressive ataxia occurring later than AOA1, characterized by increased alpha-fetoprotein levels (8, 9), (iii) *PIK3R*5 (phosphoinositide-3-kinase, regulatory subunit 5), responsible for AOA3 (MIM 615217), with clinical features similar to AOA2; (iv) *PNKP* (polynucleotide kinase 3′-phosphatase) responsible for AOA4 (MIM 616267) characterized by ataxia, oculomotor apraxia, peripheral neuropathy, and dystonia (3).

These syndromes impact cerebellar function and result in a profound loss of motor control characterized by cerebellar degeneration, apraxia, and oculomotor apraxia (abnormal saccadic eye movement). Very often these symptoms occur in the presence of dystonia and peripheral neuropathy (5, 10).

AOA4 is characterized by prominent dystonia which spontaneously attenuates during the course of the disease. Muscle wasting in the hands and feet and neuropathy are also common and lead to tetraplegia and short atrophic hands and feet. Usually, cognitive function is not affected, although some people may present intellectual disability. AOA diseases are characterized by an early onset which occurs in the first decade; strikingly, AOA4 is characterized by the earliest disease onset among AOAs which occurs at about 4 years of age (11).

*PNKP* is a DNA processing enzyme in which the C-terminal catalytic domain contains a fused bimodal phosphatase and kinase domain, with a fork-head-associated (FHA) domain at its N-terminus (12).

The FHA domain of *PNKP* is important for interaction with either the XRCC1 or XRCC4 scaffold proteins, which are required for assembling single-strand break repair (SSBR) or dominant pathway for DNA double-strand break repair (DSBR) (NHEJ, non-homologous end-joining) components respectively (13–15). In fact, *PNK*P is important for both the SSBR/Base excision repair (BER) and NHEJ pathways (12).

To date, only a few patients with mutations in *PNKP* have been reported worldwide. Substantial variation exists among affected individuals with *PNKP* mutations, as individuals diagnosed with MCSZ show no neurodegeneration, while individuals with AOA4 show pronounced neurodegeneration.

Among AO4 patients, the different phenotypes associated with mutations in *PNK*P do not seem to relate to either the type or the location of the mutation (3). So far, all *PNKP* mutations related to AOA4 have been described in the kinase domain.

In this report, we describe a patient affected by AOA4 related to a homozygous mutation in *PNKP*, with very late onset at 50 years. Despite the occurrence of AOA4 phenotype, this variant falls within the FHA domain, which was never reported before in AOA4 patients.

## Materials and Methods

### Genetic Testing

**Figure 1:**
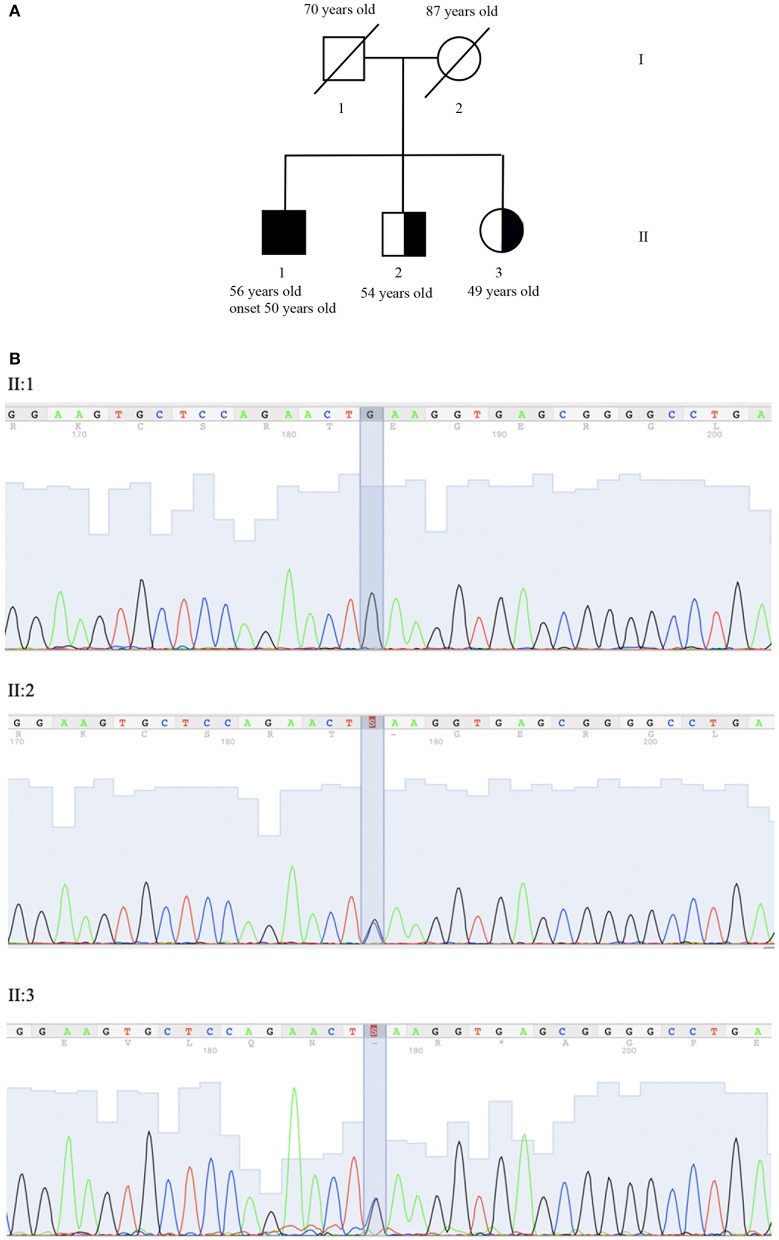
Genetic evaluation. **(A)** Pedigree: II:1 corresponds to proband, II:2 and II:3 to the asymptomatic carrier brother and sister. **(B)** Co-segregation analysis of NM_007254.3:c.[148C>G]; NP_009185.2: p. (Gln50Glu) (rs756746191:C>G) (Clin Var Accession Number SUB6349616) in exon 2 of gene *PNKP* (OMIM #605610). Sequence analysis is shown for proband (II:1) who is homozygous for variant and brother (II:2) and his sister (II:3) who is heterozygous carrier of the same variant.

The pedigree of the proband's family is shown in Figure 1A. After genetic counseling, written informed consent was obtained, and clinical exome sequencing (4,800 human genes) was performed including 100 genes related to Ataxia and or Spastic Paraplegia (Clinical Exome Solution, SOPHiA genetics) on MiSeq platform (Illumina) (16).

SOPHiA DDM (SOPHiA genetics) was used for annotation and characterization of variants. Sequence analysis identified the homozygous mutation NM_007254.3:c.[148C>G]; NP_009185.2: p. (Gln50Glu) (rs756746191:C>G).

In the FHA domain *PNKP* (OMIM #605610), resulting in the replacement of glutamine with glutamic acid in the protein at position 50. The variant was confirmed by Sanger sequencing (ABI 3130xl Genetic Analyzer, Applied Biosystem) (Figure 1B). No mutations in other ataxia-related genes were identified. The variant has been submitted to Clinical Variant (ClinVar Accession Number SUB6349616).

The frequency of the variant is not known either in the ExAC database (http://evs.gs.washington.edu/EVS) or in the 1,000 Genomes database (http://browser.1000genomes.org). The prediction analysis *in silico* reveals a damaging effect in three out of four tools used [Mutation taster (http://www.mutationtaster.org), SIFT (http://sift.jcvi.org), and Polyphen-2 (http://genetics.bwh.harvard.edu/pph2)]. The PROVEAN (http://provean.jcvi.org/index.php) tool alone predicts it as neutral. The analysis using the PhyloP (http://compgen.bscb.cornell.edu/phast/) and GERP (http://mendel.stanford.edu/sidowlab/downloads/gerp/index.html) tools gave respectively a positive score (1) and an neutral rate (NR) equal to 5.4 indicating that both the wild type nucleotide and the amino acid are conserved in mammals.

The presence of p.Gln50Glu was evaluated in II:2 (54 years old brother) and II:3 (49 years old sister), who are asymptomatic heterozygous carriers (Figure 1A).

According to ACMG guidelines, which consider data on familial segregation, bioinformatic analysis, and allele frequencies, p. (Gln50Glu) has been classified as a likely pathogenic variant.

## Results

A 56-year-old Italian male (II:1) was referred to the Neurology Unit of the Scientific Institute for Research and Healthcare Neuromed in Pozzilli (IS) Italy, with a 6 years history of distal weakness and tactile hypoesthesia of the legs and arms, cramps, urinary urgency, dysarthria, and mild dysphagia. He was born at term by unrelated parents that died late in life. His comorbidity consists of allergic bronchial asthma which was routinely treated with beta_2_-agonists and corticosteroids (Figure 1A).

**Figure 2:**
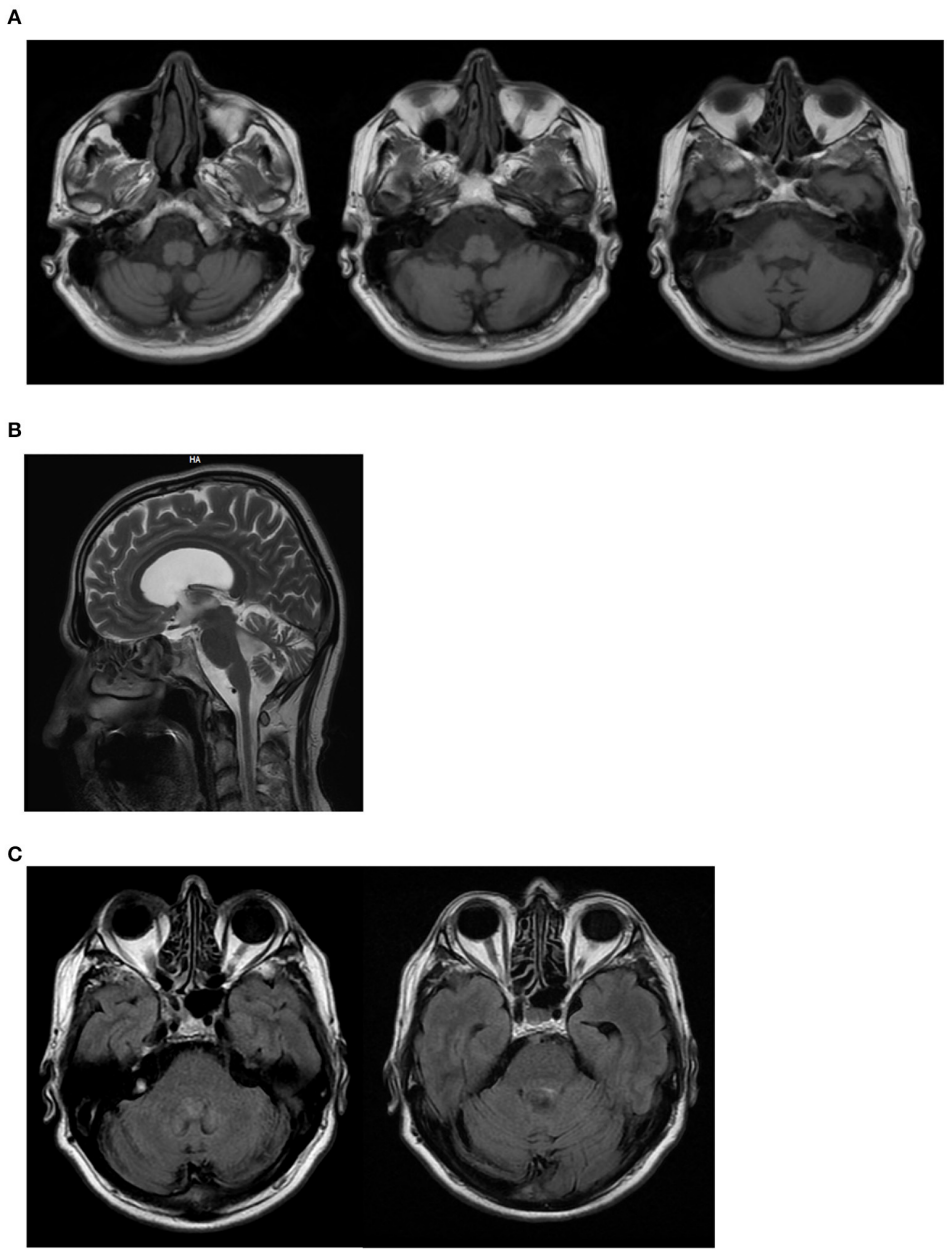
II:1 Brain MRI. **(A)** Axial T1-weighted shows cerebellar and brainstem atrophy; **(B)** Sagittal MRI scan shows global cerebellar atrophy; **(C)** Axial FLAIR MRI sequences shows no dentate nucleus hyperintensity.

On neurological examination, he had cerebellar dysarthria, gait ataxia (the patient was able to walk without support but with moderate gait instability), mild bilateral dysmetria, oculomotor apraxia, distal weakness of the arms and legs, distal hypoesthesia, absent tendon reflexes. The International Cooperative Ataxia Rating Scale (ICARS) total score was 40/100. Electroneuromyography (ENMG) indicated the presence of a sensory-motor axonal polyneuropathy. Motor evoked potentials (MEPs) were characterized by increased central motor conduction time. Somatosensory evoked potentials (SEP) were not measurable due to a lack of reproducibility of peripheral responses. Brain Magnetic Resonance Imaging (MRI) at the age of 55 showed marked subtentorial atrophy without spinal cord alteration. In detail, Axial T1-weighted showed cerebellar and brainstem atrophy, Sagittal MRI scan showed global cerebellar atrophy, and Axial FLAIR MRI sequences showed no dentate nucleus hyperintensity (Figure 2). The neuropsychological assessment showed impairment of short-term memory, attention and constructive apraxia. Nothing relevant was found on body Computer Tomography (CT) scan. There were no alterations in the liquor analyzed after lumbar puncture. A second neurological examination performed after 6 months showed no significant deterioration, suggesting a slow progression of the disease.

Blood tests showed mild hyper-CKemia (624 UI/l), hypercholesterolemia (263 mg/dl), hight alfa-fetoprotein (10.85 ng/ml), and low albumin level (3.4 g/dl). BMI index was 24.22. LDL increased level (173 mg/dl, nv < 130) confirmed data by Scheiss et al. (4), who reported increased LDL and IgE levels in AOA4 patients. The immunoglobulin dosage was not performed. High-density lipoprotein (HDL), triglycerides, thyroid hormones, vitamins, autoimmune screen, anti-ganglioside, and anti-neuronal antibodies were normal.

## Discussion

A variety of human neurologic diseases are caused by inherited defects in DNA repair, underscoring the critical requirement for genome stability in this tissue (17). An example is provided by the defective enzymatic activity of the genes responsible for ataxia with AOA, a subgroup involving cerebellar ataxia, axonal neuropathy, oculomotor apraxia, and extrapyramidal features. Genes responsible for this condition provide instructions for making proteins that are involved in repairing damaged DNA. DNA damage that is not repaired makes the cell unstable and can lead to cell death, producing a severe effect in the cerebellum, as witnessed by the occurrence of ataxia with oculomotor apraxia (18).

Recently described in a Portuguese family (19), AOA4 is a complex, progressive movement disorder characterized by phenotypic heterogeneity like other AOAs. In most patients, the first symptom is dystonia, which spontaneously attenuates during the course of the disease. Other features include cerebellar ataxia, oculomotor apraxia, and polyneuropathy. Cognitive impairment is rare, while a few patients at later stages of the disease present abnormal values of alfa-fetoprotein, albumin, and cholesterol.

In this report, we describe the later onset of AOA4 in a patient that did not experience dystonia but a classic cerebellar syndrome with gait ataxia, mild dysmetria, oculomotor apraxia, and cerebellar dysarthria and dysphagia. Sensorial-motor axonal polyneuropathy was also present. Cerebellar ataxia was compatible with cerebellar atrophy detected at brain MRI (20) (Figure 2). Dysphagia could be explained by the involvement of the nucleus ambiguous in the brainstem atrophy detected at MRI. However, considering the concomitance of cerebellar dysarthria, the presence of dysphagia is likely to be due to the cerebellar involvement. Similarly, brainstem atrophy could be further investigated concerning the potential involvement of pre-cerebellar nuclei. Mild hyper-CKemia, although non-specific, is likely to be due to the motor component of polyneuropathy.

The AO4 patient here described is unique considering his age of onset at 50. In fact, age of onset of AO4 patients ranges from 1 to 9 years (with a mean of 4.3). So far, only a German woman was described with a delayed onset although she was much younger (23-year-old), compared with the present case report. Additionally, this 23 year old diagnosed patient possessed an AOA phenotype which was combined with a cerebellar pilocytic astrocytoma (21).

The present paper reports a patient who is a carrier of the homozygous mutation NM_007254.3:c.[148C>G]; NP_009185.2: p. (Gln50Glu) (rs756746191:C>G) in the FHA domain PNKP. To date, multiple distinct human syndromes have been linked to mutations in *PNKP*, leading to microcephaly or neurodegeneration (17). *PNKP* is a multifunctional DNA repair enzyme important for both the SSBR/BER and NHEJ pathways (12), and this dual functionality potentially explains the presence of both microcephaly and neurodegeneration in patients with certain inherited *PNKP* mutations (22).

In support of this, it's well-known that different domains of the protein are linked to different diseases. The kinase domain of *PNKP* is associated with neurodegeneration associated with AOA4, as witnessed by the variants p.G375W, p.T408del, p.R439fs, p.T442fs, p.Thr424fs*49, and p.Tyr515* (21). Mutations responsible for MCSZ are mainly located in the phosphatase domain (p.L176F and p.E326) but spread along the whole gene. In detail, an MCSZ patient carries the compound heterozygote genotype p.G292R/p.A55S, falling respectively in the phosphatase and FHA domains; while another MCSZ patient is homozygous for p.T424GfsX48 in the kinase domain (a variant which was also reported in a compound heterozygous AOA4 patient) (2, 23).

Remarkably, so far variants in the FHA domain have never been associated with AOA4 and even with neurodegeneration or microcephaly, as they have been solely reported in individuals who manifest only seizures (p.P20S, p.A55S) (11).

The genetic analysis performed in this paper has limitations. In fact, although the sequencing analysis has been carried out on 4,800 human genes including 100 genes related to Ataxia and Spastic paraplegia, these can't rule out the involvement of other genes related to clinical phenotype. Probably, a more extensive approach like whole exome or whole genome sequencing would guarantee more informative data. Moreover, segregation analysis is not fully informative. In fact, the age of the brother and the sister of the proband, both asymptomatic heterozygous carriers, can't rule out the appearance of clinical symptoms in the next years.

The present paper describes the first variant in the FHA domain leading to neurodegeneration and appearing with an AOA4 phenotype. Further studies are required to establish the role of mutations in different PNKP domains with a special emphasis of site-specificity within the FHA domain, and to identify and understand the role of modifier genes (24). This is required to explain the molecular mechanisms leading to the onset of AOA4 phenotype and to understand whether a late disease onset is a typical feature of AOA4 when this is produced by FHA domain specific mutations.

## Author Contributions

SG and RF interpretation of data and drafting of manuscript. FF and DC acquisition of data, analysis and interpretation of data, and critical revision. RC, RF, FBi, and MC acquisition of data, analysis and interpretation of data. FBu and CF neurological history and interpretation of clinical data. MM, EG, and AR analysis and interpretation of data and critical revision. SG study conception and design, analysis and interpretation of data, and drafting of manuscript.

### Conflict of Interest

The authors declare that the research was conducted in the absence of any commercial or financial relationships that could be construed as a potential conflict of interest.
